# Impact of tissue sampling on accuracy of Ki67 immunohistochemistry evaluation in breast cancer

**DOI:** 10.1186/s13000-016-0525-z

**Published:** 2016-08-30

**Authors:** Justinas Besusparis, Benoit Plancoulaine, Allan Rasmusson, Renaldas Augulis, Andrew R. Green, Ian O. Ellis, Aida Laurinaviciene, Paulette Herlin, Arvydas Laurinavicius

**Affiliations:** 1 Faculty of Medicine, Vilnius University, M.K.Ciurlionio 21, Vilnius, LT-03101 Lithuania; 2 National Center of Pathology, affiliate of Vilnius University Hospital Santariskiu Clinics, P. Baublio 5, Vilnius, LT-08406 Lithuania; 3 PathImage/BioTICLA, Inserm (UMR 1199), University Caen Normandy, Cancer Center F. Baclesse, Caen, France; 4 Division of Cancer and Stem Cells, School of Medicine, University of Nottingham, Nottingham, UK; 5 Histopathology, Nottingham City Hospital University of Nottingham, Nottingham, UK

**Keywords:** Tissue microarrays, TMA, Digital image analysis, Breast cancer, Ki67, Tumor heterogeneity, Tissue sampling

## Abstract

**Background:**

Gene expression studies have identified molecular subtypes of breast cancer with implications to chemotherapy recommendations. For distinction of these types, a combination of immunohistochemistry (IHC) markers, including proliferative activity of tumor cells, estimated by Ki67 labeling index is used. Clinical studies are frequently based on IHC performed on tissue microarrays (TMA) with variable tissue sampling. This raises the need for evidence-based sampling criteria for individual IHC biomarker studies. We present a novel tissue sampling simulation model and demonstrate its application on Ki67 assessment in breast cancer tissue taking intratumoral heterogeneity into account.

**Methods:**

Whole slide images (WSI) of 297 breast cancer sections, immunohistochemically stained for Ki67, were subjected to digital image analysis (DIA). Percentage of tumor cells stained for Ki67 was computed for hexagonal tiles super-imposed on the WSI. From this, intratumoral Ki67 heterogeneity indicators (Haralick’s entropy values) were extracted and used to dichotomize the tumors into homogeneous and heterogeneous subsets. Simulations with random selection of hexagons, equivalent to 0.75 mm circular diameter TMA cores, were performed. The tissue sampling requirements were investigated in relation to tumor heterogeneity using linear regression and extended error analysis.

**Results:**

The sampling requirements were dependent on the heterogeneity of the biomarker expression. To achieve a coefficient error of 10 %, 5–6 cores were needed for homogeneous cases, 11–12 cores for heterogeneous cases; in mixed tumor population 8 TMA cores were required. Similarly, to achieve the same accuracy, approximately 4,000 nuclei must be counted when the intratumor heterogeneity is mixed/unknown. Tumors of low proliferative activity would require larger sampling (10–12 TMA cores, or 6,250 nuclei) to achieve the same error measurement results as for highly proliferative tumors.

**Conclusions:**

Our data show that optimal tissue sampling for IHC biomarker evaluation is dependent on the heterogeneity of the tissue under study and needs to be determined on a per use basis. We propose a method that can be applied to determine the sampling strategy for specific biomarkers, tissues and study targets. In addition, our findings highlight the benefit of high-capacity computer-based IHC measurement techniques to improve accuracy of the testing.

**Electronic supplementary material:**

The online version of this article (doi:10.1186/s13000-016-0525-z) contains supplementary material, which is available to authorized users.

## Background

Gene expression studies have identified distinct molecular subtypes of breast cancer (Luminal A, Luminal B, HER2-enriched, basal-like and normal breast-like) with markedly different behavior and prognosis [1]. Meanwhile, clinical practice of decision making largely relies on the definition of Luminal A-like and Luminal B-like disease, based on a combination of estrogen receptor (ER), progesterone receptor (PgR) and Ki67 immunohistochemistry (IHC) [2]. Proliferative activity of tumor cells, estimated by Ki67 labeling index (Ki67 LI) is a key indicator to support this stratification and provides strong prognostic and predictive information on response to chemotherapy [3]. Clinical utility of Ki67 LI is hampered by the lack of robust measurement methodologies and widely acknowledged issue of intratumor Ki67 heterogeneity expression. Consequently, it is hard to achieve consensus on cut-off values to stratify the patients for therapeutic decisions [2]. Great effort has been made to standardize the techniques for manual and digital/automated Ki67 LI measurement, including criteria for tissue sampling, hotspot detection, and digital image analysis (DIA) tools [4–11].

Recently, Ki67 expression across distinct categories of breast cancer specimens including whole slide surgical specimens, needle core biopsies and tissue microarrays (TMA) was investigated by Knutsvik et al. [1]. They found significant differences of Ki67 LI estimates across the different sample categories and suggested that specimen-specific cut-off values should be applied for practical use. While the recommendation is logical and may compensate for the inherent differences of the tissue sampling, its implementation requires better knowledge of measurement accuracy that can be achieved by the techniques, in general. Additionally, Going [12] has previously pointed out that the counting rules depend on level of mitotic activity in tumors. This dependency has not been investigated for tumors with varying Ki67 proliferation rates.

TMA has been often applied for discovery and clinical studies of IHC biomarkers. Initially proposed by Battifora [13], it enables multiple testing on numerous tissue samples in a standardized, tissue-sparing, and high-throughput manner by assembling small core biopsies from morphologically representative areas of tissues onto a single paraffin block [14]. The approach was further refined into to a precise technique by Kononen [15]. One inherent drawback of the TMA technique is related to the limited fraction of the original sample included, raising the need to achieve/be aware of adequate sampling requirements [16]. Furthermore, TMA sampling requirements may vary depending on the target, lesion, tissue, and the goal of investigation. Therefore, it is important to determine the sampling parameters on a per-use basis. For instance, three cores of 0.6 mm diameter will have almost a similar area to one core of 1 mm diameter (0.85 mm^2^ versus 0.78 mm^2^), but provide different information about the specimen as they are likely to represent multiple areas [17, 18]. To address this issue, many studies have been performed to determine the impact of size and number of TMA cores [17, 19–28]. Most commonly, the recommended number of TMA cores varied from one to four with a diameter between 0.6 mm to 2 mm.

Determining optimal TMA sampling parameters by physical sampling of the cores, is not only time-consuming, but, more importantly, it limits the options of comprehensive statistical modeling and decomposes the original tissue sample to be used as the reference standard. To overcome these limitations, the concept of a virtual TMA was explored by utilizing digital whole slide images (WSI) to extract artificial TMA cores [25]. The approach has been applied in several studies: Quintayo et al. [19] manually marked core positions on a low magnification image before acquiring images of the TMA cores at high magnification; they also matched core positions between H&E and IHC staining of the same tissue before the acquired cores were subsequently assembled to a virtual TMA of ductal carcinoma *in situ*. Pedersen et al. [28] reported a similar procedure, but used random sampling of six 1 mm diameter cores directly on 20x magnification images of both H&E and IHC slides before assembling the virtual TMA. The studies supported the principle that assembling a set of virtual TMAs by copying cores from digital images is a valuable approach in TMA-based tissue sampling modeling.

A methodology for comprehensive IHC evaluation with appraisal of intratumoral heterogeneity aspects in WSIs of Ki67-stained breast cancer tissue was recently proposed [29]. It is based on systematic subsampling of DIA-generated data into a hexagonal tiling (HexT) arrays and enables computation of a comprehensive set of texture and distribution indicators for Ki67 intratumoral variability. While the primary aim of that study was to investigate intratumoral heterogeneity of Ki67 expression, in the current study we exploit the method for modeling tissue sampling precision in homogeneous and heterogeneous tumors dichotomized by spatial entropy of Ki67 expression: The hexagons in the HexT were chosen to simulate virtual TMA cores (or corresponding fields of view in conventional microscopy), with numbers of Ki67 positive and negative cells established by DIA. Using the spatial entropy extracted from the tiling as a spatial modeling of the Ki67 expression the impact tissue and cell sample size and tumor heterogeneity has on the accuracy of Ki67 LI measurement becomes possible to investigate. We present evidence that tumors with lower Ki67 LI as well as higher spatial heterogeneity of Ki67 expression require relatively larger sampling subsets to represent the global average of the biomarker expression in the tissue. Additionally, the results support the notion, that tumors at the low end of proliferation scale require higher cell counts [12].

## Methods

### Tissue and data

A data set consisting of primary breast cancer from 297 patients was used in this study. Details of the dataset are reported in [29]. Briefly, 91 % of the tumors were invasive ductal carcinoma of the breast (270/297). Tissue samples were formalin-fixed, processed with standard paraffin embedding techniques. IHC for Ki67 was performed with antibody (clone MIB-1; DAKO, Glostrup, DK) and multimer technology-based detection system (ultraView Universal DAB, Ventana, Tucson, AZ, USA). Digital WSIs were recorded using a ScanScope XT Slide Scanner (Leica Aperio Technologies, Vista, CA, USA) under 20x objective magnification (0.5-μm resolution) and subsequently subjected to DIA by the Leica Aperio Genie Classifier v.1/Nuclear v.9 algorithm. This tool was previously calibrated based on tumor versus benign tissue recognition and positive versus negative cells detection. DIA algorithm was previously validated using a criterion standard achieved by stereological counting. The research was approved by the Vilnius Regional Biomedical Research Ethics committee (reference number NR.:40, date 2007-04-26). Additional informed consent was not required for the use of archived material.

### TMA simulation using hexagonal tiling

**Fig. 1:**
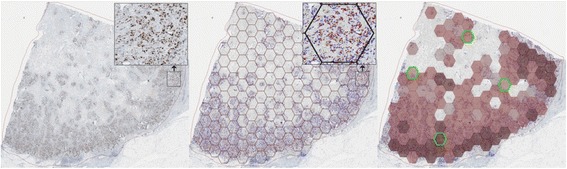
Hexagonal tiling of digital image analysis data for tissue subsampling simulations. Left: Tumor marked by region of interest. Overlay showing high resolution tissue. Middle: Tumor with results of DIA and the hexagonal grid for TMA simulation. Overlay showing high resolution DIA results. Right: Hexagonal grid filtered according to nuclei count. Ki67 LI indicated by fill color. Light gray is low Ki67 LI with darker reds showing larger Ki67 LI. Green hexagons illustrate one possible subsampled set of four hexagons

The HexT methodology forming the basis of automated texture feature extraction is described in detail in [29]. Briefly, the coordinates of positive and negative nuclei extracted by DIA were distributed into a dense HexT overlaid on each WSI. The HexT was randomly positioned within the invasive tumor area (Fig. 1, Middle). Hexagons containing no nuclear profiles by DIA were regarded as missing data; hexagons containing fewer than 100 nuclear profiles were regarded as insufficiently sampled. A minimum requirement of 30 informative hexagons per tumor was applied. Local Ki67 LI was calculated for each hexagon to construct co-occurrence matrix used to compute Haralick texture parameters.

The individual hexagons, with local Ki67 LI, were subsequently used as TMA cores for the random sampling simulations (Fig. 1, Right) and resembled approximately a TMA core of 0.75 mm circular diameter and 0.44 mm^2^ area. The tumors were dichotomized into homogeneous and heterogeneous groups based on the median entropy value obtained by the HexT methodology. The sampling simulations were carried out for all three tumor classes: all/mixed, homogeneous and heterogeneous.

In addition to giving insight about the minimum number of required TMA cores, the simulations can be used to infer error measurements according to how many nuclei are assessed. By dichotomizing the simulated cores by the number of nuclei contained, the error measurement can additionally be investigated as function of the nuclei count.

### The experimental models and statistical methods

The impact caused by varying core number was investigated for a range of numbers feasible to punch out in practice. The chosen set of core numbers investigated is denoted HexN = (1, 2, …, 15).

The practical evaluation of Ki67 LI scores from multiple cores or tissue regions is not always based on individual cell counts. Here we investigated the impact of three ways of calculating the Ki67 LI from a set of subsampled virtual cores: mean, median and by first summing total numbers of positive and negative nuclei in the subsampled hexagons, denoted sum. For a subsampled set H the Ki67 LI by sum is simply:

$$ \mathrm{sum}(H)\kern0.5em =\kern0.5em \frac{\underset{i\kern0.5em =\kern0.5em 1}{\overset{HexN}{\varSigma }}\mathrm{P}\mathrm{o}\mathrm{s}\left( he{x}_i\right)}{\underset{i\kern0.5em =\kern0.5em 1}{\overset{HexN}{\varSigma }}\mathrm{P}\mathrm{o}\mathrm{s}\left( he{x}_i\right)\kern0.5em +\kern0.5em \underset{i\kern0.5em =\kern0.5em 1}{\overset{HexN}{\varSigma }}\mathrm{N}\mathrm{e}\mathrm{g}\left( he{x}_i\right)}, $$

where Pos and Neg are functions counting positive and negative nuclei in a hexagon, respectively. Note that if TMA cores could sample the entire area of the tumors, only the evaluation by “sum” would be equivalent to the Ki67 LI determined by whole slide image analysis which extracts all nuclei before calculating Ki67 ratio.

Two different methods were used to simulate the impact of the number of hexagons/TMA cores on the precision of the sampling to represent the Ki67 LI reported by the DIA of the entire region of interest (ROI). First, the practice of “physical” TMA construction, in which a set of cores is sampled only once, was simulated by randomly sampling a subset of hexagons once. Single linear regression analysis was used to compare the data in a single random selection.

Secondly, an error analysis was conducted by simulating many samplings of TMA subsets with core numbers of sizes HexN = (1, 2, …, 15) per case. Each subset is sampled from the set of hexagonal tiles without replacement, but all hexagons are replaced before sampling a new subset. From the resulting sampling distribution, error measurements and other statistics can be inferred. Here, the simulations were used to infer the coefficient of error (CE) of Ki67 LI predictions using subsets differing in the number of virtual cores. The CE was calculated as

$$ CE\kern0.5em =\kern0.5em \sqrt{\frac{Bia{s}^2\kern0.5em +\kern0.5em {\sigma}^2}{T^2}}\kern0.5em =\kern0.5em \sqrt{\frac{{\left(\mu -\kern0.5em T\right)}^2\kern0.5em +\kern0.5em {\sigma}^2}{T^2}}, $$

where σ is standard deviation, μ is mean of Ki67 LI inferred from the simulation distributions and T is the Ki67 LI as determined by the DIA. The interpretations of error analysis results are made according to a putative CE value of 10 % for accessible results for practical applications. The choice of this value is strongly influenced by CE dependence on Ki67 LI heterogeneity levels (Haralick entropy values). This dependence is illustrated in additional plots available as Additional file 1.

Both experiments were grouped by tumor heterogeneity and repeated for HexN = (1,2, …,15) with hexagons resembling a 0.75 mm diameter TMA core and the simulations were performed with 50,000 iterations.

From the simulations error measurements according to how many nuclei are assessed can be inferred as follows: for one tumor case 50,000 subsets of TMA cores are sampled of size HexN = (1, 2, …, 15). This yields a total of 750,000 subsets which are effectively grouped by HexN. By dichotomizing according to the number of nuclei sampled in each subset into bins of 250 nuclei (first bin [0;250), second bin [250;500) etc.), the error measurement can additionally be investigated as function of the nuclei count. To make it clear if CE is calculated according to hexagon area or nuclei number, the CE is denoted *CE*_*Area*_ and *CE*_*Nuclei*_*,* respectively.

Previously, Going [12] pointed out that to achieve the same relative error large cell counts are required for low mitotic activity tumors while high mitotic activity requires more moderate cell counts. Specifically, it was illustrated that the relationship between the relative error in the mitotic activity can be approximated by $Relative\kern0.5em  Error\kern0.5em \approx \kern0.5em \frac{1}{\sqrt{n}}\kern0.5em =\kern0.5em {n}^{-0.5}$, where n is number of mitoses. Here we investigate if a similar relationship exists between relative error measurements *CE*_*Area*_ and *CE*_*Nuclei*_ as function of the Ki67 proliferation activity indicator by fitting CE as function of Ki67 to

$$ CE\kern0.5em =\kern0.5em a\kern0.5em {x}^{-b}. $$

This is done for each choice of HexN, for a set of bins used for dichotomizing by nuclei count and for all three classes of heterogeneity (all/mixed, homogeneous and heterogeneous).

Statistical analysis was performed using R 3.1.2, GNU GCC 5.2.1, Open Office 4.1.2 and SAS 9.4 software.

## Results

### Summary statistics

Extensive dataset summary statistics of the Ki67 indicators, obtained by HexT methodology, are previously reported [29]. Briefly, the global average of Ki67 LI values (in percentages) estimated by DIA of the WSIs was almost identical to the results obtained by HexT (mean: 32.5 ± 16.9 %, median 32.6 ± 17.4 % and sum 32.7 ± 17.3 %). Importantly, the HexT data provided a comprehensive set of intratissue variation indicators [29].

### Single subsampling – linear regression analysis

**Fig. 2:**
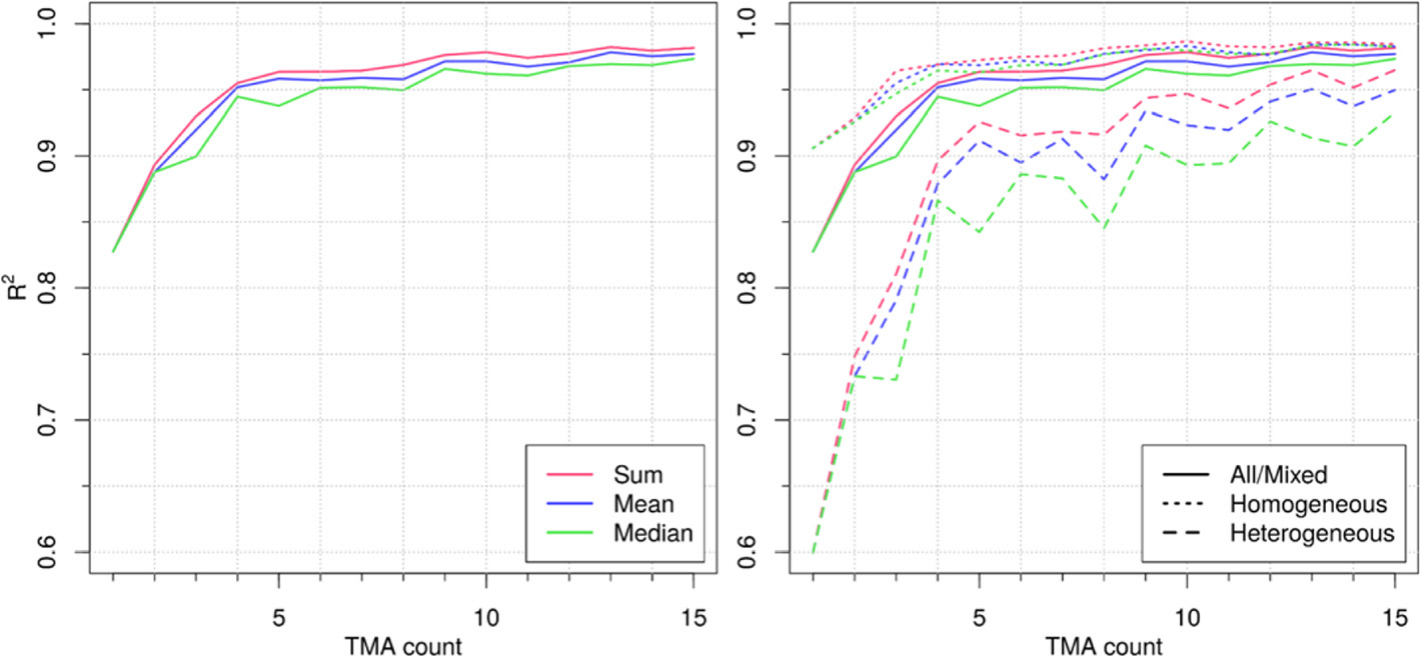
Linear regression results for single random selection. Linear regression analysis results for hexagon size = 825 pixels ≈ 0.75 mm TMA core. Ratios by sum, median and mean were used on a subset of hexagons. Results are divided by tumor heterogeneity. Note that y-axis begins at R^2^ = 0.6 for better visualization of differences between groups of measurements

Figure 2 illustrates the linear regression analysis results (R^2^ values) plotted by different types of Ki67 LI calculation methods (mean, median, and sum) and grouped by heterogeneity. All R^2^ values from linear regression analysis were at *p* < 0.0001 significance level. The R^2^ values for all cores are presented in Table 1. Linear regression analysis (Fig. 2) reveals that R^2^ values plotted for various Ki67 LI measurement methods were nearly overlapping in the subgroup of homogeneous tumors. For the heterogeneous tumors, mean and median were less representative than the sum-based percentage. This bias was mostly apparent with small sets of cores and diminished when a larger number of cores were used.

**Table 1:** Linear regression analysis results for hexagon size = 825 pixels (≈0.75 mm TMA core)

R^2^ values									
HexN	All tumor cases			Homogeneous cases			Heterogeneous cases		
	Sum	Mean	Median	Sum	Mean	Median	Sum	Mean	Median
1	0.827	0.827	0.827	0.906	0.906	0.906	0.6	0.6	0.6
2	0.893	0.888	0.888	0.929	0.926	0.926	0.749	0.733	0.733
3	0.93	0.92	0.9	0.964	0.955	0.947	0.81	0.79	0.731
4	0.955	0.952	0.945	0.969	0.969	0.965	0.897	0.879	0.866
5	0.964	0.958	0.938	0.972	0.969	0.963	0.926	0.912	0.842
6	0.964	0.957	0.951	0.975	0.972	0.969	0.916	0.895	0.886
7	0.964	0.959	0.952	0.976	0.969	0.969	0.918	0.913	0.883
8	0.969	0.958	0.95	0.981	0.977	0.977	0.916	0.882	0.845
9	0.976	0.971	0.966	0.984	0.98	0.981	0.944	0.934	0.908
10	0.978	0.971	0.962	0.987	0.983	0.98	0.947	0.923	0.893
11	0.974	0.968	0.961	0.983	0.978	0.977	0.936	0.92	0.894
12	0.977	0.971	0.968	0.982	0.976	0.977	0.954	0.941	0.926
13	0.982	0.978	0.969	0.986	0.984	0.984	0.965	0.95	0.914
14	0.98	0.975	0.969	0.986	0.984	0.984	0.952	0.938	0.907
15	0.982	0.977	0.973	0.984	0.983	0.983	0.965	0.95	0.933

In each data set Ki-67 LI was calculated by counting mean, median and sum of positive and negative cells. All linear regression analysis results were statistically significant, *p* < 0.0001

To achieve R^2^ = 0.95 value in the regression models, random selection of at least four, three and twelve cores were required in the mixed, homogeneous and heterogeneous tumors, respectively.

### Error analysis

**Fig. 3:**
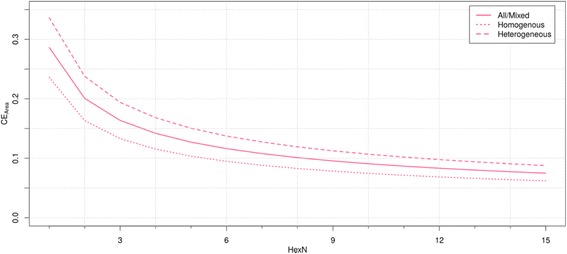
Error results as function of tissue area evaluated. The resampling procedure was simulated for each individual tumor case using 50,000 iterations for each count of hexagons (HexN). Analysis results are split by tumor heterogeneity level. Error measurement (Coefficient of error) is expressed by mean of all cases

The mean coefficient of error (CE) for Ki67 LI estimates, calculated using the sum, is plotted for increasing TMA core numbers in the tumor subgroups (Fig. 3). To achieve the CE of 10 %, 8 cores of 0.75 mm diameter were required in the mixed group of tumors. Respectively, 5–6 or 11–12 cores were required in the subgroups of homogeneous and heterogeneous tumors.

**Fig. 4:**
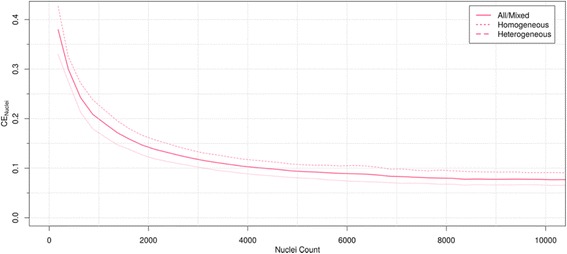
Error results as a function of nuclei counted. The coefficient of error plotted as a function of nuclei count. See text for transformation of TMA by core number to nuclei count. Analysis results are split by tumor heterogeneity level. Error measurement (Coefficient of error) is expressed by mean of all cases

To achieve a CE of 10 %, approximately 4,000 nuclei were required in the mixed group of tumors as depicted in Fig. 4. For the subgroups of homogeneous and heterogeneous tumors to reach the same error, 3,000 and 7,000 nuclei were necessary, respectively.

**Fig. 5:**
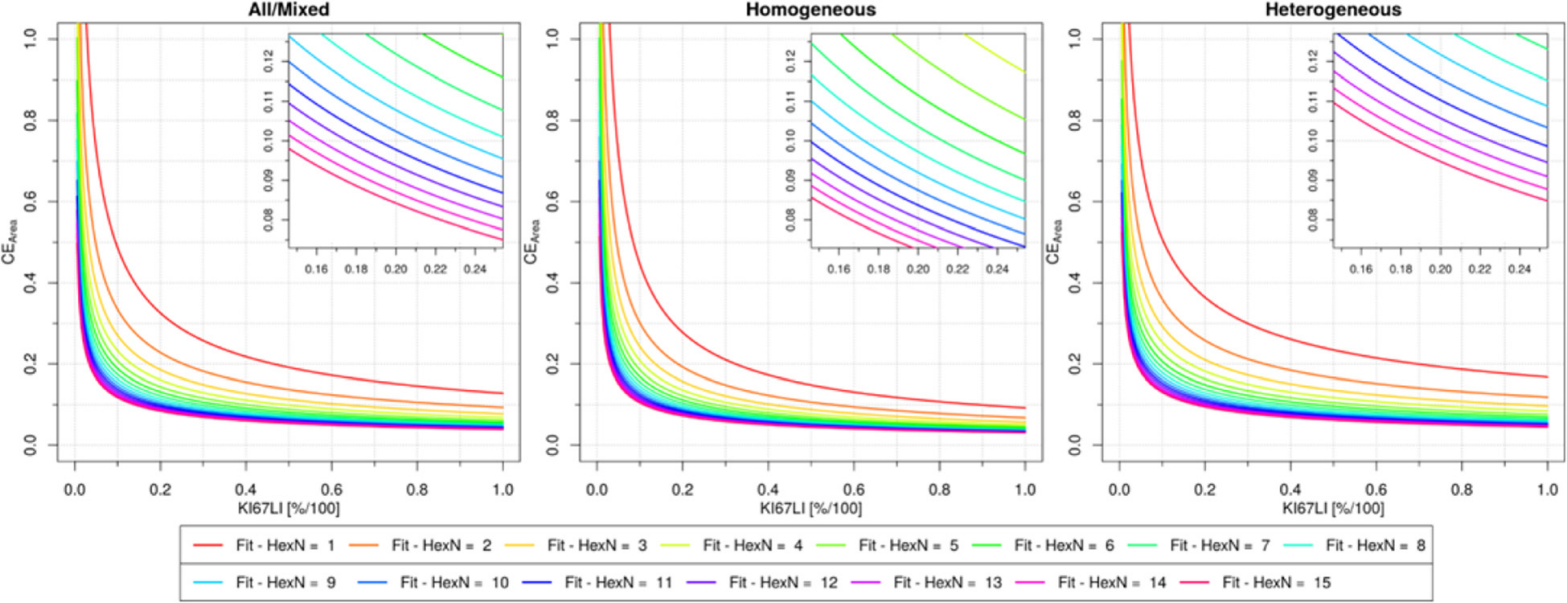
Coefficient of error by tissue area evaluated as a function of Ki67 LI in tumors of different heterogeneity level. CE_Area plotted as depending on heterogeneity level with a separate curve for each HexN = (1,…,15). See Additional file 1 for curve fits

An inverse relationship between CE_Area and proliferation activity is clearly seen in Fig. 5 for any choice of TMA count. Furthermore, Table 2 reveals that the fitted parameter b is close to the value of 0.5 as reported in [12] confirming the same dependence. The close-up around the critical point of 10 % CE and 20 % Ki67 LI in Fig. 5 shows that for mixed tumor population to achieve the CE of 10 %, approximately 10–11 TMA cores were required at the level of 20 % Ki67 LI. Respectively, 7–8 or 13–14 cores were required in the subgroups of homogeneous and heterogeneous tumors.

**Table 2:** Fit parameters for relative error CE_Area fitted to proliferation index for all three heterogeneity classes

Proliferation fit to relative error						
HexN	All/Mixed		Homogenous		Heterogeneous	
	a	b	a	b	a	b
1	0.128	0.58	0.092	0.684	0.168	0.481
2	0.093	0.553	0.068	0.647	0.119	0.482
3	0.077	0.542	0.057	0.63	0.097	0.48
4	0.068	0.533	0.051	0.614	0.084	0.478
5	0.062	0.527	0.046	0.604	0.076	0.477
6	0.057	0.521	0.043	0.595	0.069	0.474
7	0.053	0.516	0.04	0.587	0.064	0.473
8	0.05	0.512	0.038	0.579	0.06	0.473
9	0.048	0.507	0.037	0.572	0.057	0.471
10	0.046	0.502	0.035	0.564	0.054	0.469
11	0.044	0.498	0.034	0.557	0.052	0.469
12	0.042	0.493	0.033	0.549	0.05	0.467
13	0.041	0.489	0.032	0.543	0.048	0.463
14	0.04	0.486	0.031	0.538	0.047	0.463
15	0.039	0.482	0.031	0.531	0.045	0.464

**Fig. 6:**
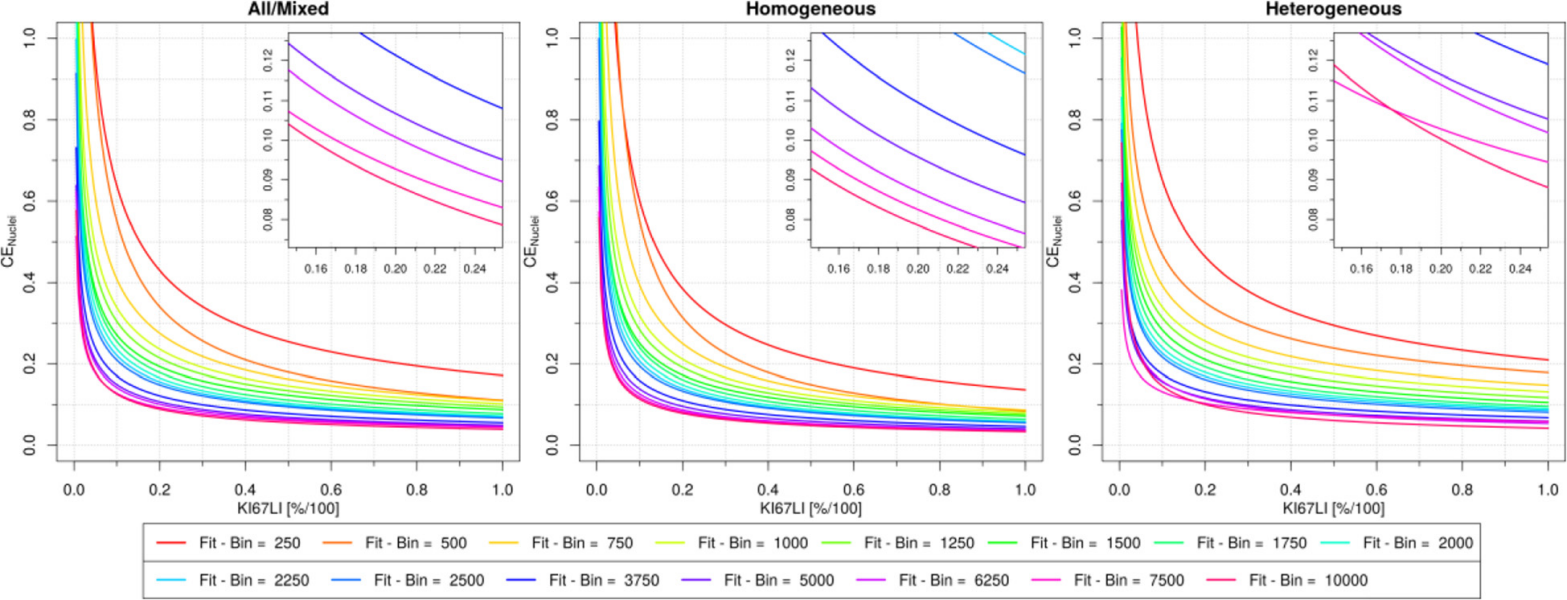
Coefficient of error by nuclei counted as a function of of Ki67 LI in tumors of different heterogeneity level. CE_Nuclei plotted as depending on heterogeneity level with a separate curve for a selected subset of nuclei bins. See Additional file 1 for curve fits

Similarly, a high CE_Nuclei is also observed for low proliferation rates as depicted in Fig. 6. which graphically confirms the need for counting more nuclei for low proliferation tumors. Also here the fitting parameter b is close to the value 0.5 reported [12], see Table 3. The close-ups in Fig. 6 reveal that at the level of 20 % Ki67 LI, to achieve the CE of 10 %, approximately 6,250 nuclei were required in the mixed group of tumors. For the subgroups of homogeneous and heterogeneous tumors to reach the same error 5,000 and 10,000 nuclei were necessary, respectively.

**Table 3:** Fit parameters for relative error CE_Nuclei fitted to proliferation index for all three heterogeneity classes

Proliferation fit to relative error						
Nuclei bin	All/Mixed		Homogenous		Heterogeneous	
	a	b	a	b	a	b
250	0.171	0.571	0.137	0.644	0.209	0.494
500	0.111	0.697	0.085	0.815	0.178	0.421
750	0.109	0.573	0.087	0.658	0.148	0.425
1000	0.102	0.522	0.081	0.595	0.132	0.412
1250	0.094	0.509	0.076	0.572	0.117	0.426
1500	0.087	0.491	0.072	0.544	0.105	0.43
1750	0.078	0.516	0.064	0.575	0.096	0.434
2000	0.072	0.516	0.058	0.58	0.09	0.425
2250	0.069	0.504	0.056	0.567	0.086	0.419
2500	0.067	0.493	0.055	0.547	0.081	0.426
3750	0.055	0.487	0.046	0.538	0.068	0.411
5000	0.049	0.485	0.041	0.534	0.059	0.422
6250	0.046	0.494	0.037	0.539	0.054	0.47
7500	0.044	0.464	0.035	0.526	0.058	0.356
10000	0.039	0.507	0.033	0.532	0.042	0.543

## Discussion

This study has exploited novel opportunities that digital microscopy images offers for virtual TMA modeling with incorporation of DIA results. Firstly, the virtual TMAs were modeled after the HexT methodology extracted both global texture information and local feature information from the WSI. Secondly, simulation of the TMA cores using the HexT dataset enabled multiple random sampling iterations bypassing the digital assembly of the virtual TMAs. This gave a much greater flexibility in investigating a wider range of sampling methodologies, parameters and error measurements. The added benefits do not impose any new limitations: if cores are needed for several stainings of the same tissue, cores can be sampled at the exact location in different images by applying mapping techniques similar to the ones reported by Quintayo et al. [19].

Previously, a similar approach was tested by Heus et al. [30], who utilized a dense grid of rectangular frames instead of hexagons. From each subsampled frame, a core was simulated by the largest circle contained within. This has a side-effect that tissue located at the corners of the frames will never be sampled; this effect is not independent of size of the simulated cores. The use of hexagons for virtual core simulation does not suffer from this: the dense HexT ensures that all parts of the tissue are considered with the same probability. Sampling without replacement further ensures that the same area is not represented by multiple cores in the subsets used in the simulations.

The analysis of core/cell sampling requirements in this study was made possible to group according to Haralick entropy texture feature extracted by the HexT methodology for each WSI. It must be noted that the Haralick entropy threshold value is not clearly defined. Therefore, the optimal method to split the dataset it into equal parts by median was chosen. In a similar study, the variance of the local Ki67 LI was used as entropy measurement, but without a complete error analysis for the entire dataset [30].

Combining Ki67 LI (or any other biomarker) from several cores is often needed in TMA studies. This introduces a risk of bias which involves assessing the number of positive and negative nuclei for the observed cores before recalculating the Ki67 LI. We evaluated this potential bias by comparing results from combined Ki67 LI from a set of simulated cores using the mean, the median and Ki67 LI calculated by using sum of nuclei in the sampled hexagons. We found that when larger set of cores were used, any bias with regard to the Ki67 LI calculation methods was negligible (Table 1), while calculation of the combined Ki67 LI, by assessing the core data first, is strongly advised where only a few TMA cores are used from heterogeneous tumors (Fig. 2, right).

The practical TMA construction, where cores were randomly chosen only once, was investigated using linear regression. This allowed comparison of the hexagonal simulation data to previous studies. For a tumor set with mixed heterogeneity, we found a number of cores to achieve R^2 = 0.95, to be four, in line with the previous reports [17, 25, 26, 31]. For homogeneous tumors, the optimal number of cores was three, depending whether the sum or mean calculations were used, respectively. A rather dramatic increase to the requirement of 12 cores was found in the heterogeneous tumors.

The single sampling brings some eventuality, because for each sampling, it is possible to obtain cores containing different tissue representation and thus biomarker expression level. The error is particularly important when considering tissue samples with varying degrees of heterogeneity, as it influences the representativeness of TMAs [27]. A number of studies have shown that more cores will improve the agreement level and reduce the limitations due to the heterogeneity in various types of tumors and IHC biomarkers [25–27, 32, 33]. However, only the introduced method allows inference about the relative error caused by different TMA sampling parameters in combination with tumor heterogeneity. Our results show that to obtain a CE of 10 % the necessary number of cores in the dataset with mixed heterogeneity is 8; five cores hold sufficient information for Ki67 LI determination in homogeneous tumors, while heterogeneous tumors need at least 11–12 cores to be sampled.

In practical TMA applications, intratumoral heterogeneity of biomarker expression is usually unknown in advance; therefore, a more conservative approach would assume that all tumors in the study population are heterogeneous. On the other hand, Ki67 LI expression in breast cancer tissue is known for its spatial heterogeneity and may serve a reference standard for other biomarkers and tumors. In that sense, our study reveals that 11–12 random TMA cores of 0.75 mm diameter would sufficiently represent IHC biomarker expression in heterogeneous tumors. Our simulations also indicate that disagreements between different studies of TMA core numbers may in fact be due to unestablished differences in heterogeneity aspects. In general, our findings support the notion that heterogeneity information is crucial for optimizing TMA studies. Ideally, the presented method could be used in pilot studies to validate the optimal number of cores, or at least heterogeneity should be investigated from a larger set of cores, for instance by measuring a range of Ki67 LI between several TMA cores taken from the tissue.

Our study also provides evidence for minimum cell counting requirements to achieve robust Ki67 LI measurement, especially with regard to the limited capacity of manual counting procedures. Current clinical guidelines on the minimal number of cells to be counted are quite arbitrary, mostly set in the range of 500 and 2000 tumor cells [9]. While small samples (e.g., needle core biopsies) may allow counting all the invasive tumor cells, it becomes impractical in larger samples. Therefore, to achieve adequate precision, it is recommended for the interpreting pathologist to score at least 1,000 cells, while 500 cells would be acceptable as the absolute minimum [9]. Importantly, our findings reveal that to achieve 10 % CE approximately 4,000 nuclei must be counted when the intratumor heterogeneity is mixed/unknown (Fig. 4). These cell counts are rather large to accomplish in clinical practice for all breast carcinomas, but could be feasible for cases considered as “grey zone”, e.g. in the range of Ki67 LI 10-30 % [3]. A visual scoring methodology proposed by Hida et al., might be used as method of choice for “low” (Ki67 LI <10 %) or “high” (Ki67 LI >30 %) proliferatively active cases, leaving behind “grey zone” cases, which requires more precise methodologies [34].

The inverse relationship between relative estimation error and mitotic activity previously highlighted by Going [12] was confirmed to also exist between each of the two error estimates (*CE*_*Area*_, *CE*_*Nuclei*_) and the Ki67 LI proliferation activity indicator (Figs. 5 and 6). This dependency of CE on Ki67 LI shows that tumor cases with low proliferation rate contribute most of the CE in Fig. 4 which is a set of “mixed” proliferation rate. Consequently, when scoring a single case with unknown Ki67 LI one may need to evaluate a higher cell count or larger TMA sample to ensure a 10 % CE at a specific grey zone. Specifically, the tumors at the lower scale of proliferative activity (Ki67 LI < 20 %, Fig. 5, left) will for a mixed/unknown heterogeneity case require larger sampling (at least 10–11 TMA cores) to achieve the same error measurement (10 % *CE*_*Area*_) results as for highly proliferative tumors (4–6 TMA cores). Similarly, for cases with Ki67 LI < 20 % (Fig. 5, right) at least 6,250 nuclei are necessary (for 10 % *CE*_*Nuclei*_) As such, Figs. 5 and 6 may aid determining practical sampling requirements of individual cases for acceptable CE at specific grey zones.

In general, the results of our study suggest that adequate accuracy levels of Ki67 LI measurement can hardly be achieved by manual counts and argue in favor of DIA-based techniques to benefit from the high-capacity methods. In addition, automated hotspot detection with standard definitions by DIA, which was out of scope in the present study, would provide another advantage compared to the visual evaluation by conventional microscopy or inspection of WSI.

## Conclusion

Several aspects raised in this study relate to the evaluation of Ki67 immunohistochemistry in breast cancer in clinical research and practice. Firstly, obtaining an optimal number of TMA cores/cell number needed for biomarker research studies depends on the tissue, especially its intratissue heterogeneity and level of expression. For Ki67 LI in breast cancer, we found 5–6 cores sufficient for homogeneous expression in the tissue, 8 cores for tumors with mixed heterogeneity and at least 11 cores for heterogeneous tumors. Secondly, our findings reveal that to achieve low error estimates when evaluating by cell counting, approximately 4,000 nuclei must be evaluated when the intratumor heterogeneity is mixed/unknown. In breast cancer cases of the lower proliferative activity (Ki67 LI < 20 %) larger sampling is required to achieve the same error measurement results as for highly proliferative tumors. The presented data may aid in defining practical sampling requirements of individual cases and specific grey zones.

The wide range of the number of cores/nuclei needed supports the notion that optimal sampling requirements must be determined on a peruse basis and that heterogeneity information must be assessed in the study. The method presented can be applied for individual pilot study measurements. In addition, our findings highlight the importance of high-capacity computer-based IHC measurement techniques to improve accuracy of the testing.

## Abbreviations

CE, coefficient of error; *CE*_*Area*_, coefficient of error calculated according to hexagon area; *CE*_*Nuclei*_:coefficient of error calculated according to nuclei number; DIA, digital image analysis; ER, estrogen receptor; H&E, hematoxylin and eozin staining; HexN, set of hexagonal cores; HexT, hexagonal tiling; IHC, immunohistochemistry; Ki67LI, Ki67 labeling index; PgR, progesterone receptor; TMA, tissue microarray; TMAs, tissue microarrays; WSI, whole slide images

## Additional files

Additional file 1: Fits curves of proliferation index to CE_Area and CE_Nuclei (depending on heterogeneity levels). Graphs for CE plotted as a function of Area (Hexes in case). (DOCX 12 mb) Additional file 2: Initial dataset of the study. (XLSX 2 mb)
