# Supplementary material for: Impact of tissue sampling on accuracy of Ki67 immunohistochemistry evaluation in breast cancer
Source: Diagn Pathol. 2016 Aug 30;11(1):82. doi: 10.1186/s13000-016-0525-z (PMC5006256; doi:10.1186/s13000-016-0525-z)

# Additional file 1

## Fits of proliferation index to CE_Area

## All/Mixed group
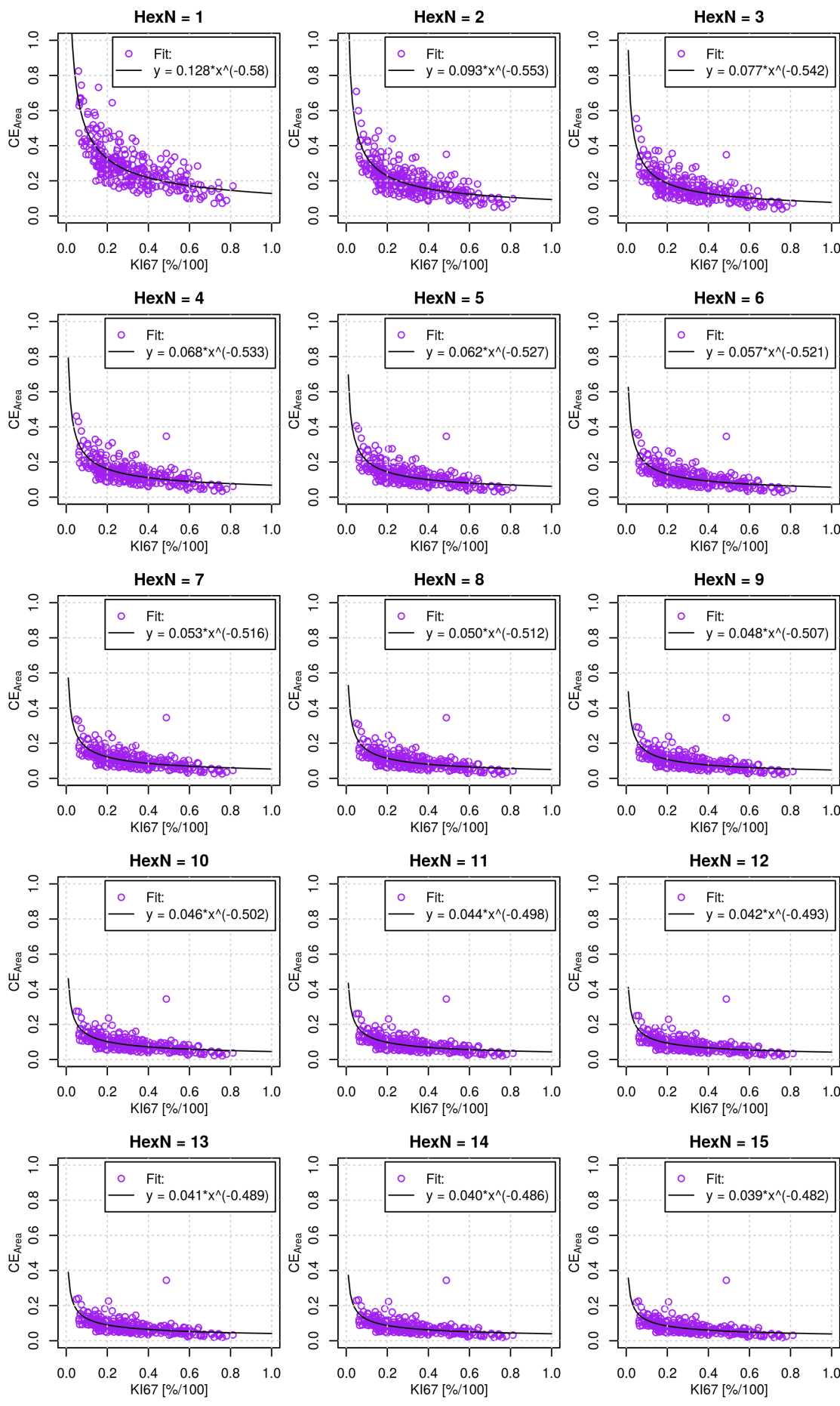


### Homogeneous group


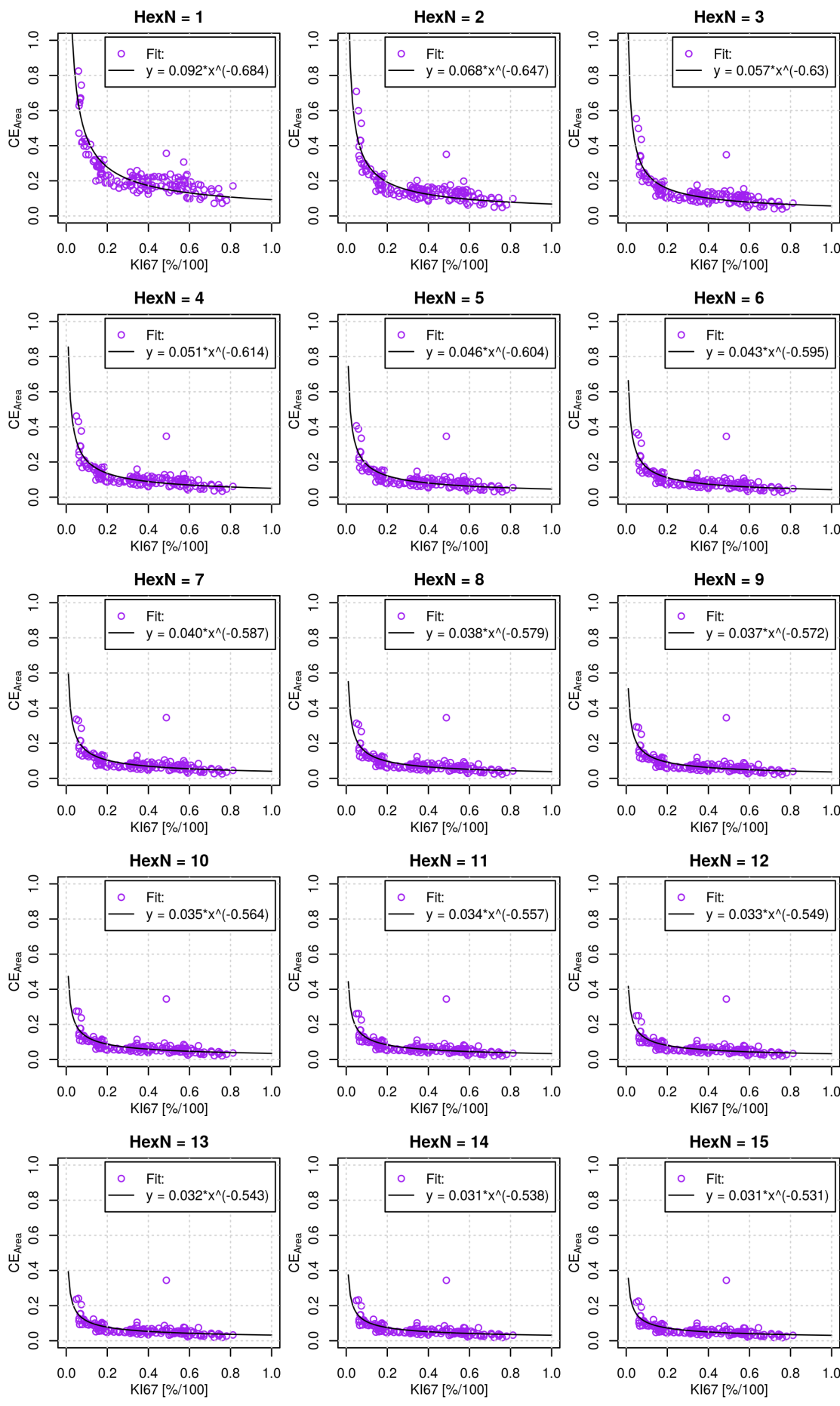


### Heterogeneous group


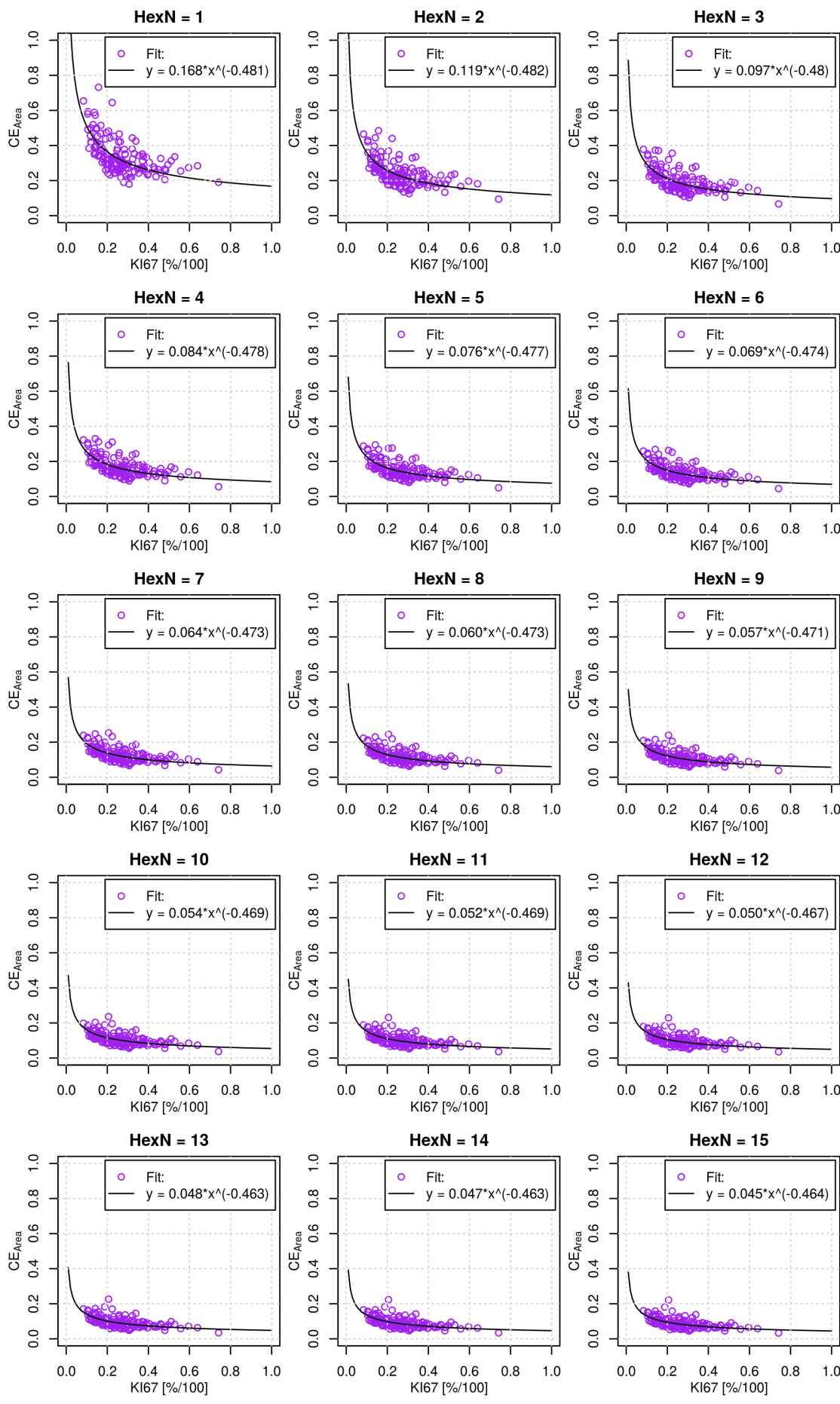


## Fits of proliferation index to CE_Nuclei

### All/Mixed group


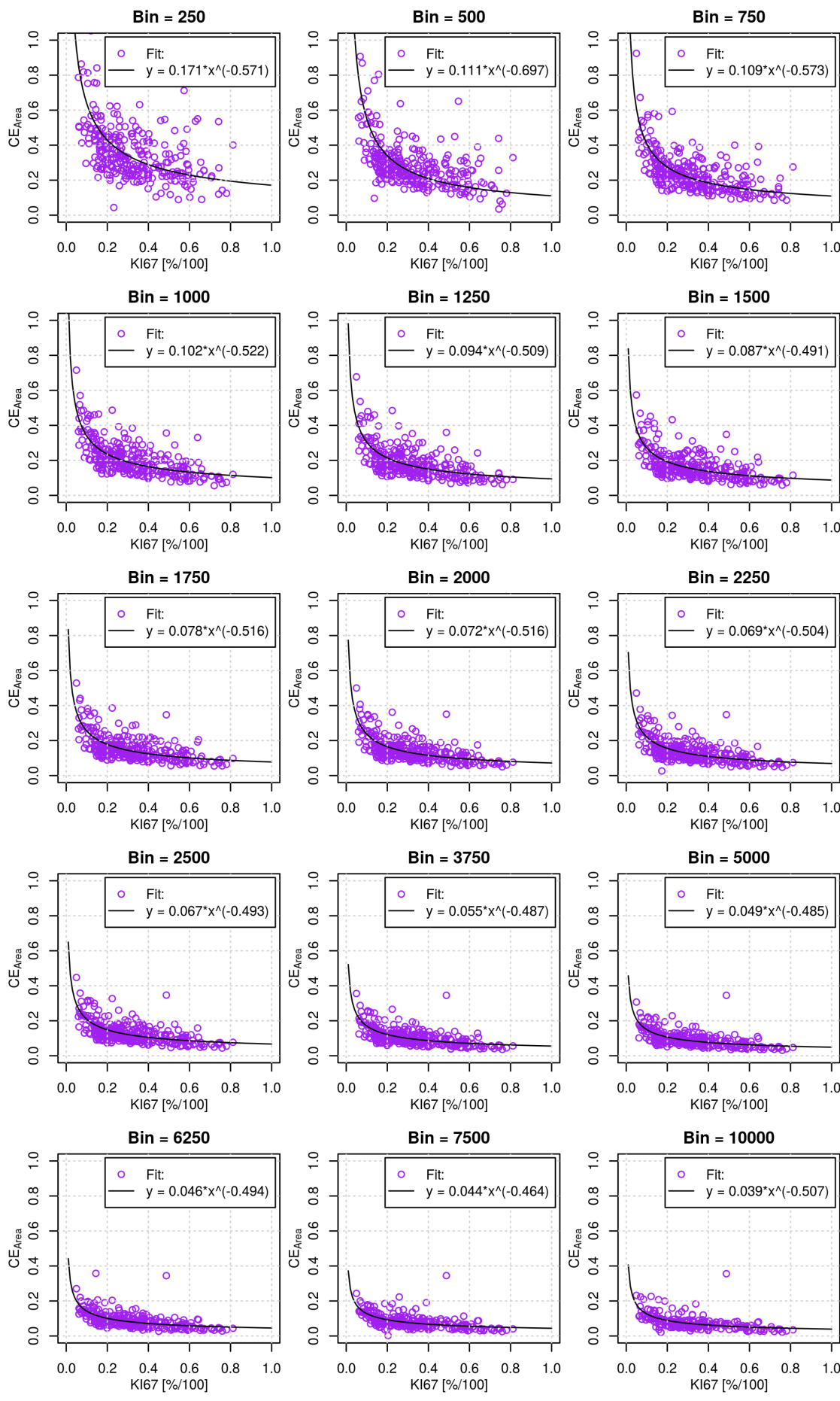


### Homogenous group


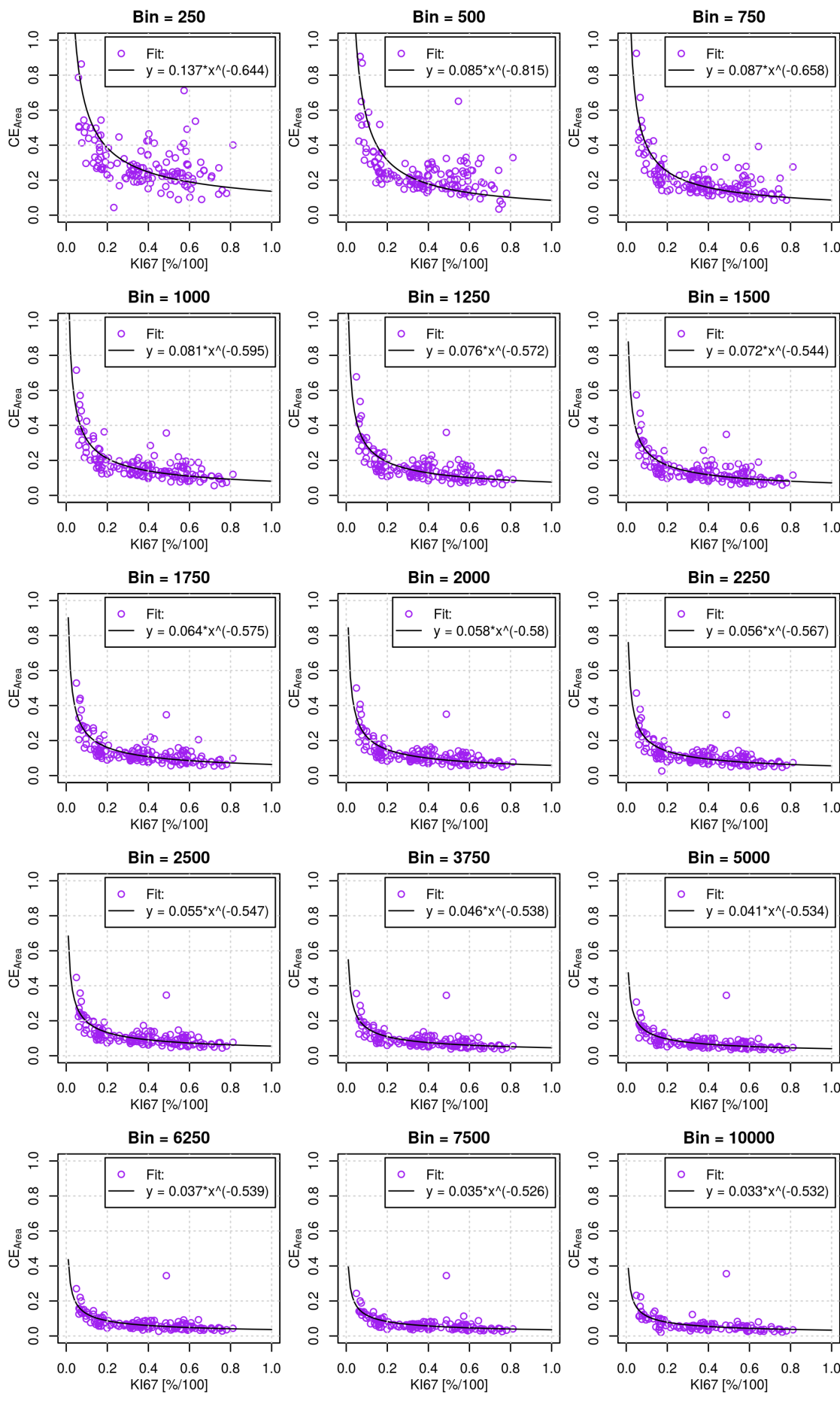


### Heterogeneous group


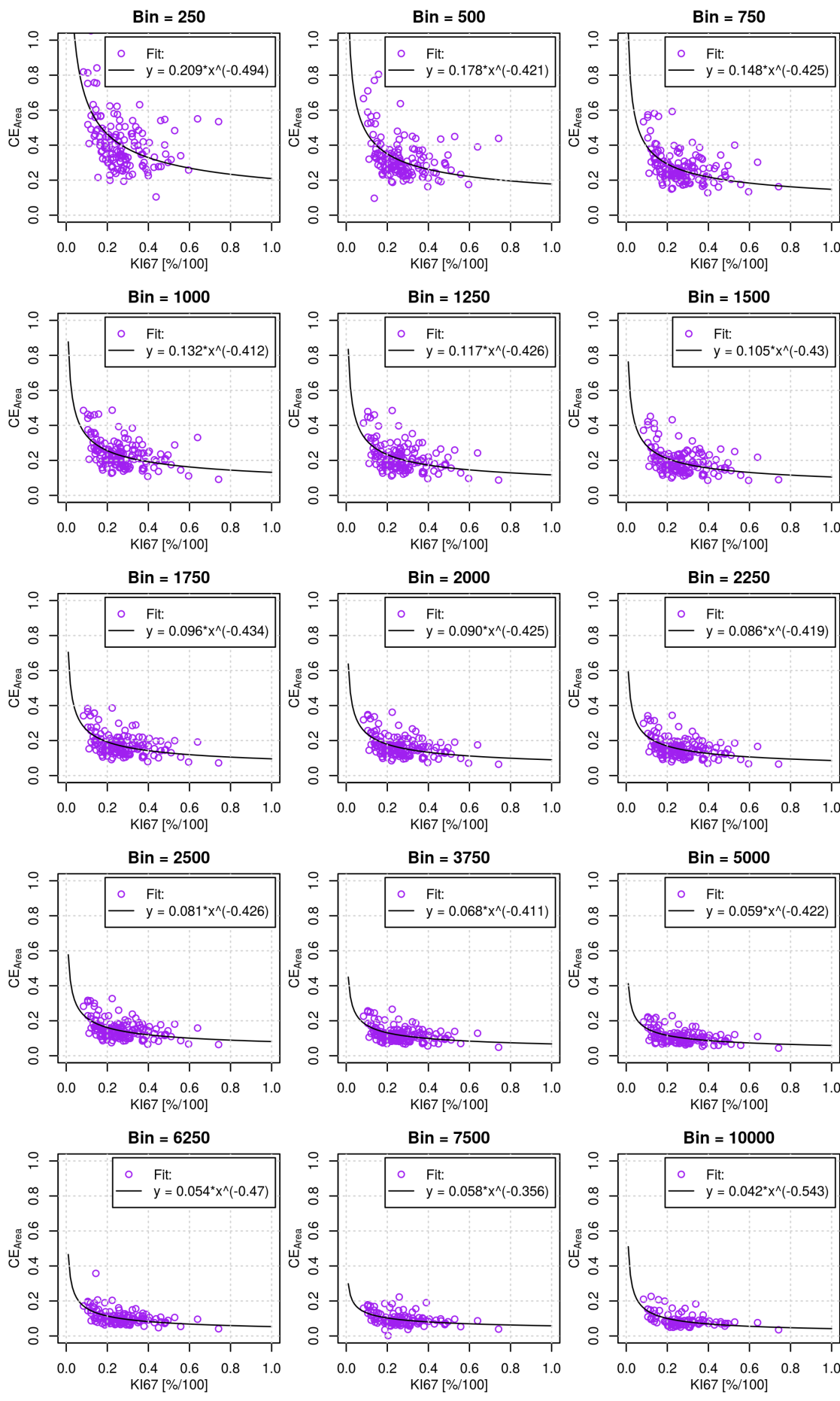


# CE plotted as function of Area (Hexes in Case)

### CE calculated over 50000 iterations for each case.

### Horizontal lines indicate 10% and 5% CE.


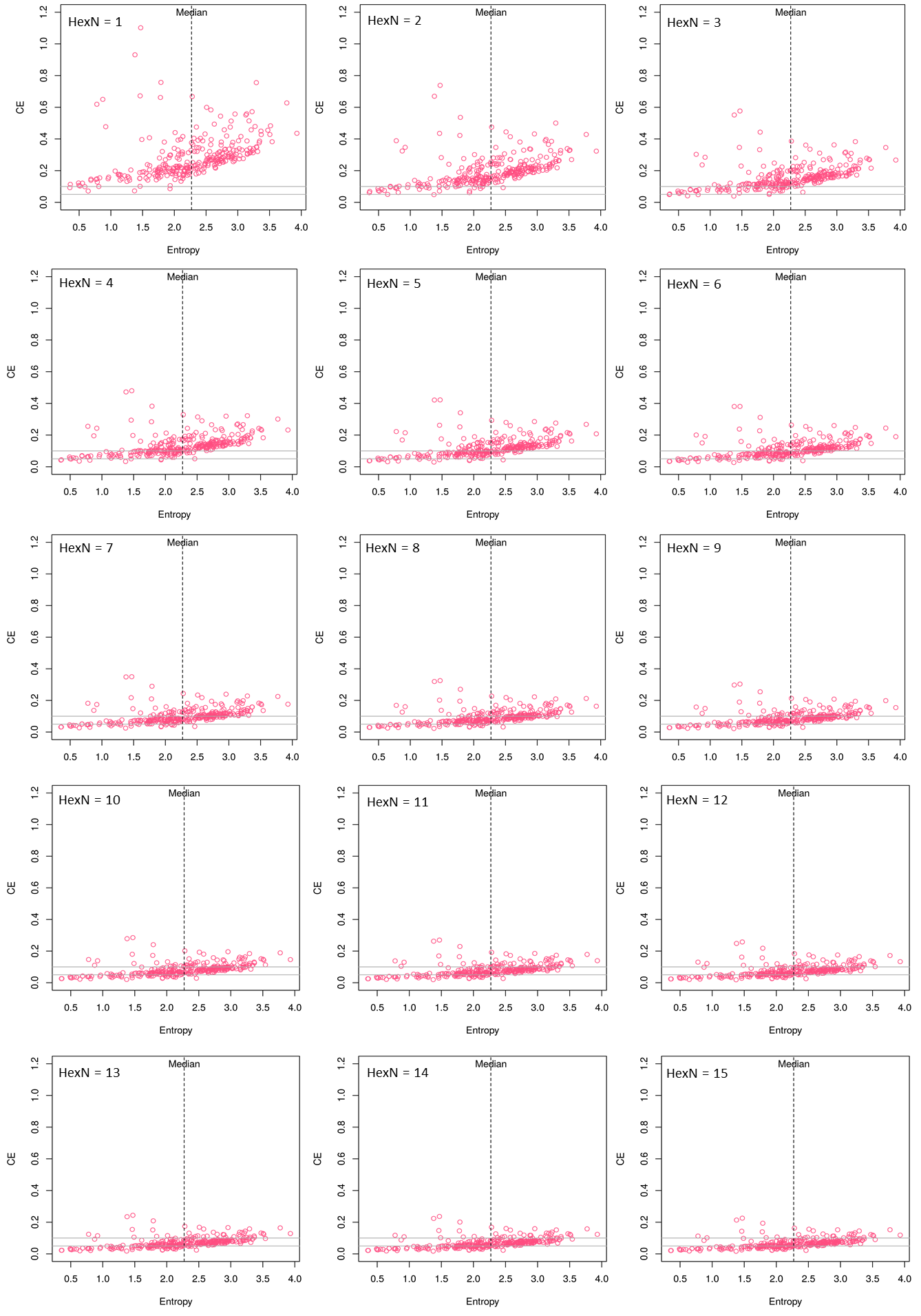

Supplement: Additional file 1: — Fits curves of proliferation index to CE_Area and CE_Nuclei (depending on heterogeneity levels). Graphs for CE plotted as a function of Area (Hexes in case). (DOCX 12 mb) [file 13000_2016_525_MOESM1_ESM.docx]
